# Minor acidic glycans: Review of focused glycomics methods

**DOI:** 10.1016/j.bbadva.2025.100150

**Published:** 2025-02-16

**Authors:** Keita Yamada

**Affiliations:** The Laboratory of Toxicology, Faculty of Pharmacy, Osaka Ohtani University, 3-11-1 Nishikiori-kita, Tondabayashi, Osaka, 584-8540, Japan

**Keywords:** Sulfated glycan, Phosphorylated glycan, Glucuronylated glycan, HNK-1 epitope, Anion exchange chromatography, HILIC, α-dystroglycan

## Abstract

Glycans are modified by acidic molecules, including sialic acid, sulfates, phosphates, and glucuronic acid, forming acidic glycans. Among these, sialylated glycans form major components of deuterostome glycomes, and their structure and function have been widely studied. The other acidic glycans, comprising minor components of the glycome, are often overlooked by glycomics and glycoproteomics methods, although they are implicated in conditions such as inflammatory diseases, neurological diseases, and viral infections. Therefore, minor acidic glycans are high-priority targets for glycomics, and analytical techniques focused on minor acidic glycans are being developed. This review examines the methods of enriching the minor acidic glycans from biological glycomes and examines their structure, providing examples of the application of these techniques in biological and clinical samples.

## Highlights

• Acidic glycans participate in inflammation, infection, and neurological diseases.
• We review methods to analyze sulfated, phosphorylated, and glucuronidated glycans.
• Methods to enrich minor acidic glycans are evaluated.
• Methods to distinguish sulfated from phosphorylated glycans are discussed.
• This review summarizes advances and state-of-the-art techniques in glycomics.

## Graphical abstract

**Figure:**
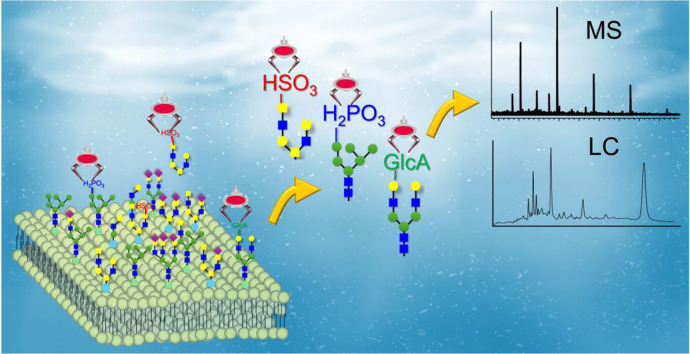

## 1 Introduction

**Fig. 1:**
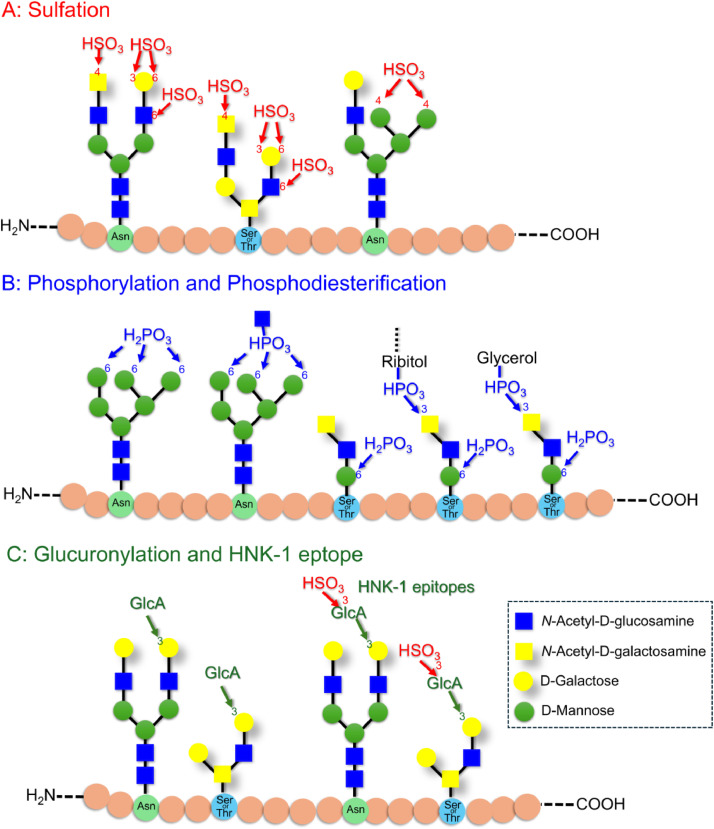
Typical minor acidic glycan structure. A) Sulfation, B) phosphorylation, and C) glucuronidation of glycans. In this illustration, the glycans are bound to glycoproteins; however, sulfation and glucuronidation can also be observed in glycolipids.

Glycans on proteins and lipids are modified by various acidic molecules, resulting in negatively charged acidic glycans. Sialic acid-modified (sialylated) glycans are the most common acidic glycans; consequently, their functions have been widely studied [[1], [2], [3]]. Acidic glycans can also be modified with sulfates, phosphates, and glucuronic acid (Fig. 1). Such acidic glycans are minor components of the biological glycome, and their expression tends to be significantly lower than that of sialylated glycans, making them difficult to detect.

In the case of sulfate modifications of vertebrate glycans, for example, a sulfate group is added to the C-3 and C-6 of galactose (Gal) or the C-6 of *N*-acetylglucosamine (GlcNAc) in asparagine-linked glycans (*N*-glycans) or serine/threonine-linked glycans (*O*-glycans) [[4], [5], [6], [7]] (Fig. 1A). Sulfate groups are added to C-4 of *N*-acetylgalactosamine (GalNAc) in LacdiNAc (GalNAcβ1–4GlcNAc-R), at the non-reducing end of glycans [8], and to the C-4 of mannose in hybrid type *N*-glycans [9]. A sulfate group can also be added to the sialic acid on the glycan [10]. Although the functions of sulfated glycans remain largely unknown, they are thought to participate in immune responses and inflammatory diseases. For example, *O*-glycans with a 6-sulfated Gal, which are highly expressed on endothelial venules, promote lymphocyte homing by binding to L-selectin [5]. Interactions between these sulfated glycans and L-selectin participate in the development of chronic gastritis and chronic colitis [11,12].

Phosphorylation occurs at the C-6 position of mannose in glycans with high mannose content that are attached to lysosomal enzymes [13] (Fig. 1B). Phosphodiester glycans, in which GlcNAc is attached to the phosphate group, also occur on lysosomal enzymes. Such phosphodiester glycans are intermediates that are converted into phosphorylated glycans by the GlcNAc-1-phosphodiester *N*-acetylglucosaminidase [14]. The resulting phosphorylated glycans participate in lysosomal enzyme transport via the M6P receptor in the Golgi apparatus [15]. In muscle tissue, phosphorylated *O*-mannosyl glycans occur on the cell surface of α-dystroglycan [16]. These phosphorylated glycans bind to the extracellular matrix and help to anchor the cells to the basement membrane. In muscular dystrophy, the biosynthesis of these phosphorylated glycans is defective, thereby weakening muscle tissue [17]. *O*-mannosyl glycans with phosphodiester groups, in which ribitol or glycerol is attached to the phosphorylated group, have been detected on α-dystroglycan [18].

Glucuronic acid occurs primarily in glycosaminoglycans [19]; however, some is also added to the non-reducing ends of glycolipids, *N*- glycans, and *O*-glycans (Fig. 1C). Many of these are sulfated; such sulfated glucuronylated glycans are known as HNK-1 epitopes. HNK-1 epitopes occur in immune cells, including NK and T cells [20], and in neural tissue, playing an important role in the formation of neural networks [21]. HNK-1 may participate in the development of neuropathy and Alzheimer's disease [22,23]. Non-sulfated glucuronylated glycans have also been identified [24]. Sulfated, phosphorylated, and glucuronylated glycans are also found in many invertebrates and some protists; however, they are not discussed extensively here.

Many of the analytical methods used in glycomics fail to detect the minor acidic glycans, owing to their low expression, and few structural and functional analyses of minor acidic glycans have been published. In addition, existing analytical methods may cause the loss of sulfate or phosphate groups during sample preparation. Therefore, novel analytical methods are required to analyze minor acidic glycans. To analyze trace amounts of minor acidic glycans in biological samples, it is necessary to develop techniques to separate and enrich such minor acidic glycans from biological glycome. Affinity chromatography using lectins or antibodies is the method of choice for fractionating glycans with specific epitopes or structures [25,26]. However, there are currently no lectins or antibodies that can comprehensively capture minor acidic glycans. Enrichment of phosphorylated glycans can be achieved using phosphoproteomics, such as using Phos-tags and metal affinity chromatography [27]. However, because the phosphate groups on glycans sometimes form phosphodiester structures with the addition of GlcNAc or ribitol [13,18], some phosphorylated glycans cannot be captured using phosphoproteomics methods. Therefore, new methods are required to enrich these minor acidic glycans.

Distinguishing between phosphorylated and sulfated glycans presents another technical challenge. The mass difference between the sulfate and phosphate groups on the glycans is extremely low (delta mass < 0.01); therefore, considerable effort is required to distinguish between them via mass spectrometry (MS), the primary analytical method for detecting trace amounts of glycans. This mass can be distinguished by FT-ICR-MS. However, it is still challenging to distinguish trace amounts between complex sulfated and phosphorylated glycans in biological samples.

Consequently, the development of methods to discriminate between the sulfate and phosphate groups on glycans is important. This review summarizes the enrichment and structural analysis methods developed for minor acidic glycans.

## 2 Pretreatment for minor acidic glycans analysis

**Fig. 2:**
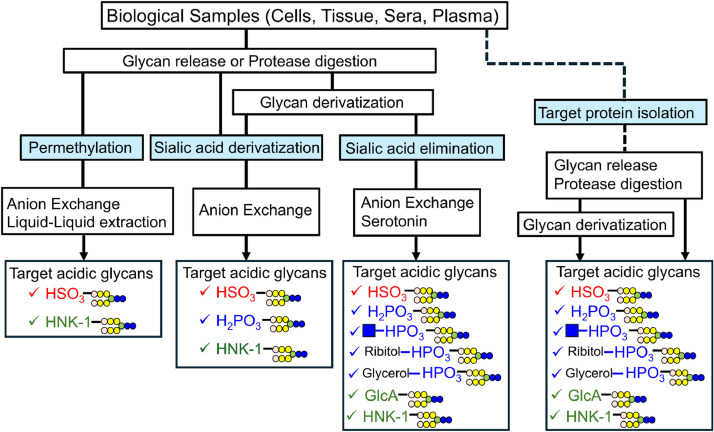
Schematic illustrating the review's focus on minor acidic glycans. Minor acidic glycans are enriched from mixtures of glycans and glycopeptides via anion-exchange chromatography and other methods. Enrichment occurs after the negative charge of sialic acid is neutralized, primarily via permethylation, sialic acid derivatization, or sialic acid elimination. Minor acidic glycans are often detected when analyzing glycans in isolated proteins. Different methods are required to detect the different types of minor acidic glycans.

Minor acidic glycans are analyzed in the same way as other glycans, by releasing the glycans from glycoconjugates such as glycoproteins and glycolipids (Fig. 2). *N*-glycans are often released from proteins via peptide *N*-glycanase F [28] or hydrazinolysis [29]. The glycans in glycosphingolipids are released from the ceramides by endoglycoceramidases (EGCase) [30]. However, EGCase is unable to release sulfated glycans from some glycolipids, such as sulfatide (HSO_3_–3Gal-Ceramide). Therefore, the analysis of sulfatides is conducted in the intact state of the glycolipids without releasing the glycans [31]. *O*-glycans, in contrast, are released via chemical release, as there are no universal endoglycosidases that can release them; they are most commonly released via alkaline β-elimination [[32], [33], [34], [35]].

The released glycans are derivatized using detection reagents, most commonly via reductive amination, hydrazine derivatization, and permethylation [36]. In some cases, glycoproteins are digested using trypsin or other peptidases and then converted into glycopeptides before analysis [37,38]. Minor acidic glycans may also be detected by comprehensive analysis of derivatized glycans or glycopeptides. The use of liquid chromatography–mass spectrometry (LC–MS) with selective ion monitoring, and the extracted ion chromatograms generated, allow for the detection of minor acidic glycans [39]. However, this method requires prior knowledge of the target structure. In addition, it is difficult to detect minor acidic glycans with low expression levels through comprehensive analysis. Therefore, enrichment is essential to analyze trace amounts of the minor acidic glycans.

Based on their negative charge, minor acidic glycans are enriched using anion exchange chromatography (AEC) and liquid–liquid extraction. As sialic acid is also negatively charged, these methods recover many sialylated glycans along with the minor acidic glycans, and these must be separated in order to enrich the minor acidic glycans. Therefore, before enrichment, the negative charge of sialic acid must be chemically neutralized, such as by permethylation or sialic acid derivatization, or the sialic acid must be removed from the glycans. LC and MS have been used to analyze minor acidic glycans can be enriched via these processes. Minor acidic glycans are sometimes detected when specific glycoproteins isolated from the proteome are analyzed. The types of minor acidic glycans that can be measured differ depending on the method used to process the sialic acid; therefore, depending on the objectives, the appropriate method must be selected (Table 1).

**Table 1:** List of examples for the analysis of minor acidic glycans.

Method	Sample	Detected glycans	Enrichment method	Ref.
Permethylation	Thyroid-stimulating hormone	Sulfated *N*-glycans	Strong anion-exchange chromatography	[42]

	Hog gastric mucin	Sulfated *O*-glycans	Mixed-mode anion exchange chromatography	[44]

	Mucin derived from human bronchial epithelial cells.	Sulfated *N*- and *O*- glycans		[43]

	Human gastric adenocarcinoma cell lines (AGS cells)	Sulfated *O*-glycans		[45]

	Human B and T cells Peripheral blood mononuclear cells	Sulfated *N*-glycans	[39]

Zebrafish	Sulfated *N*- and *O*-glycans Sulfated *N*- and *O*-glycans containing KDN *N*- and *O*- glycans containing HNK-1	[48]

Human ovarian carcinoma cells (OVCAR-3 cells)	Sulfated *N*- and *O*- glycans	[46,47]

Human salivary mucin	Sulfated *O*-glycans	Liquid–liquid extraction (water/dichloromethane)	[49]

Dipteran larvae	Sulfated *N*- and *O*-glycans Glucuronylated *N*- and *O*-glycans* O-glycans containing phosphoethanolamine		[50]

Bovine oviduct epithelial cells	Sulfated *O*-glycans	[51]

Sialic acid derivatization (Esterification)	Some glycoproteins Human saliva	Sulfated *N*- and *O*-glycans Phosphorylated *N*-glycans	Weak anion exchange chromatography	[55]

Egg whites	Sulfated *N*-glycans Phosphorylated *N*-glycans	[57]

Sialic acid derivatization (Amidation)	Bovine luteinizing hormone	Sulfated *N*-glycopeptides	Strong anion-exchange chromatography	[58]

Secreted proteins of human small cell lung carcinoma cell line	Sulfated *N*-glycopeptides	[59]

Sialic acid elimination	Human endothelial cell line(LS12 cells)	Sulfated *N*-glycans	Weak anion exchange chromatography	[62]

	Human serum	Sulfated *O*-glycans	[63]

	Some glycoproteins Gastric adenocarcinoma cell lines (MKN7 and MKN45 cells)	Sulfated *N*-glycans Phosphorylated *N*-glycans phosphodiester-type *N*-glycans	Weak anion exchange chromatography (Serotonin column)	[64]

Human serum	Sulfated *N*-glycans Phosphorylated *N*-glycans Glucuronylated *N*-glycans	[68]

Bovine submaxillary mucin porcine stomach mucin Gastric adenocarcinoma cell lines (MKN7 and MKN45 cells) Colonic adenocarcinoma cell lines (HT-29 and LS174T cells)	Sulfated *O*-glycans Sulfated *O*-glycans containing KDN Phosphorylated *O*-glycans (Phosphorylated ribose)	Weak anion exchange chromatography NH2 spin column	[67]

Target protein isolation	Porcine stomach mucin	Sulfated *O*-glycans	SMME	[69]

	Acidic mucin type glycoproteins from serous ovarian tumor cyst fluids	Sulfated *O*-glycans	Weak anion exchange chromatography	[70]

	HNK-1 carrier glycoproteins from mouse brain	*O*-glycans containing HNK-1	[71]

IgG from human serum	Sulfated *N*-glycans	Protein A immobilized Sepharose and TiO2-PGC chip	[72]

Fc-fused α-dystroglycans derived from culture medium	Phosphorylated *O*-glycopeptides Phosphodiester *O*-glycopeptides	Protein G column	[18]

⁎ In this paper, in addition to the permethylation, the glycans in Diptera larvae were analyzed using a method that combines PA labeling and fractionation using a graphite carbon column, and PA-labeled glycans containing sulfate acid and glucuronic acid were also detected.

## 3 Analysis of minor acidic glycans by permethylation

**Fig. 3:**
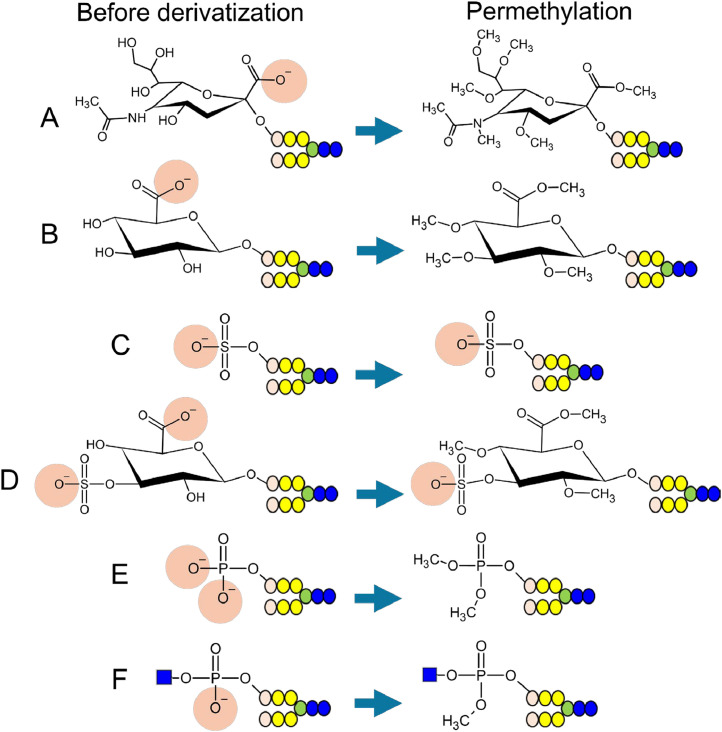
Permethylation of acidic glycans. Permethylation of (A) sialylated, (B) glucuronylated, (C) sulfated, (D) HNK-1-containing, (E) phosphorylated, and (F) phosphodiester-type glycans. Permethylation neutralizes the negative charges of glycans, except for those of the sulfate group.

Permethylation is a derivatization method used in MS analyses to improve glycan detection sensitivity [40]. Permethylation is used to derivatize alditol glycans that cannot be subjected to reductive amination and is frequently applied to analyze alditol *O*-glycans released from core proteins via reductive β-elimination. Permethylation converts the hydroxyl groups in glycans and carboxyl group of sialic acid to methyl ethers and methyl esters, respectively (Fig. 3A). It introduces methyl groups into the carboxyl groups of the glucuronic acid (Fig. 3B). Furthermore, phosphate groups (Fig. 3E and F) can be methylated but not sulfate groups (Fig. 3C and D). Therefore, in mixtures of permethylated glycans, only the permethylated sulfated glycans are negatively charged [41]. When these permethylated samples are introduced into AEC, the permethylated sulfated glycans can be captured by the stationary phase and can thus be separated from the main components of the glycome [[42], [43], [44]]. Permethylated sulfated glycans captured by AEC are eluted using a large amount of salt and are desalted using an octadecylsilyl (ODS) column or C18 ZipTip. Purified permethylated sulfated glycans are analyzed using Matrix-Assisted Laser Desorption/Ionization (MALDI)–MS and LC–MS with electrospray ionization, in both negative and positive ion modes. Many analyses of permethylated sulfated glycans in biological samples have been performed using negative ion mode. This method enables the sulfated glycans to be analyzed without the removal of sialic acid, facilitating structural analysis of acidic glycans containing sulfate and sialic acid. MS/MS analysis of permethylated glycans generates a large amount of structural information, which can be used to identify the binding position of the sulfate group. A method combining permethylation and methanolysis, which facilitates sulfate group removal from glycans, has been proposed for the structural analysis of sulfated glycans [41]. In this method, the desulfated glycans are then permethylated again using deuterium-substituted methyl iodide, and CD_3_ is introduced into the hydroxyl groups from which the sulfate groups have been removed. This method can be used to accurately determine the number of sulfate groups attached to glycans, based on the number of CD_3_ molecules introduced. Replacing the sulfate group with CD_3_ improves the detection sensitivity of these glycans in MS, facilitating highly sensitive analysis of sulfated glycans.

Permethylation has been used to analyze sulfated glycans in various biological samples (Table 1) [39]. Sulfated *N*-glycans on B cells, T cells, and human peripheral blood mononuclear cells (PBMCs) from the blood of patients with chronic lymphocytic leukemia and B-cell lymphoma were analyzed [39], and levels of permethylated sulfated *N*-glycans were determined using both MALDI–TOF (time-of-flight) MS and reverse-phase C_18_-nano LC–MS. While nano LC–MS more effectively detected permethylated sulfate glycans, MALDI–TOF MS was able to generate high-quality MS/MS spectra [39]. The same team analyzed sulfated *O*-glycans in human gastric adenocarcinoma cells, using a similar method [45]. They have detected low-sulfated keratan sulfated *O*-glycans in OVCAR-3 cells [46,47] and *N*-glycans with HNK-1 epitopes and sulfated *N*- and *O*-glycans containing 3-deoxy-D-glycero-D-galacto-2-nonulopyranosonic acid (KDN) in zebrafish organs [48].

In contrast, liquid–liquid extraction has been used to enrich permethylated sulfated glycans [49]. When permethylated glycans are mixed with water and dichloromethane (DCM), the solution separates into aqueous and DCM layers. The negatively charged sulfated glycans are collected in the water layer (the upper layer), whereas the neutral glycans and sialylated glycans that have lost their charge are collected in the DCM layer (bottom layer). The aqueous layer containing the permethylated sulfated glycans is then injected into a C18 Sep-Pak column for purification. After purification, the permethylated sulfated glycans are analyzed via electrospray ionization MS with nanospray ionization MS. This method has been used to analyze the sulfated glycans in various species of dipteran larvae [50], revealing the presence of both HNK-1 epitope and sulfated glycans. The same method was used to analyze sulfated *O*-glycans in bovine oviduct membrane glycoproteins, revealing the presence of sulfated *O*-mannose glycans [51].

Although permethylation can enrich sulfated glycans, phosphorylated glycans and non-sulfated glucuronylated glycans are recovered in the same fractions as the sialylated and neutral glycans, making it difficult to detect trace amounts of phosphorylated or non-sulfated glucuronylated glycans. In addition, since permethylation may be incomplete, sialylated glycans may be mixed with the sulfated glycan fraction.

## 4 Analysis of minor acidic glycans by sialic acid derivatization

**Fig. 4:**
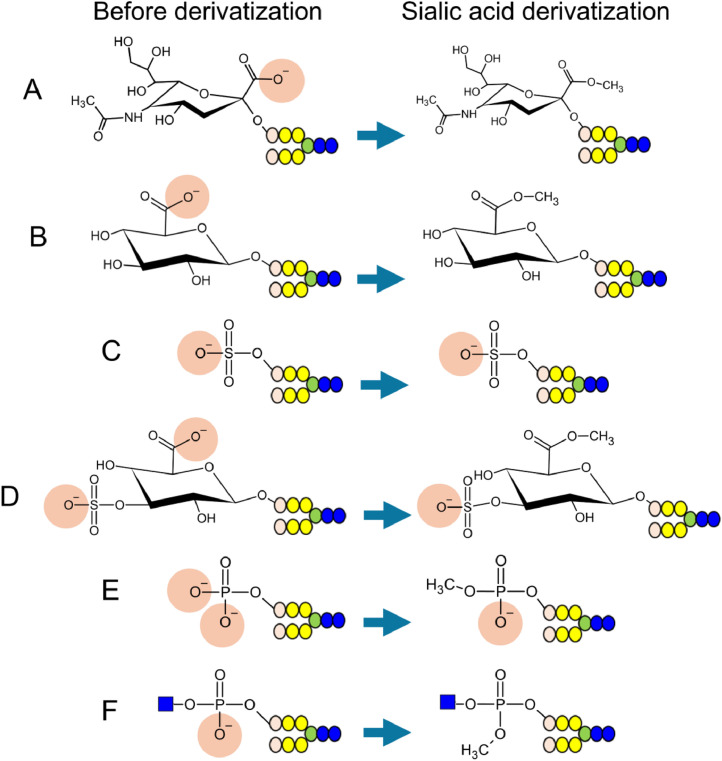
Sialic acid derivatization of acidic glycans. Methyl esterification of (A) sialylated, (B) glucuronylated, (C) sulfated, (D) HNK-1-containing, (E) phosphorylated, and (F) phosphodiester-type glycans. Methylation of the acidic glycans neutralizes the negative charges of sialic and glucuronic acid (A, B). As with permethylation, sialic acid derivatization does not methylate the sulfate group (C, D). Only one methyl group is introduced into the phosphate group, leaving a negative charge (E). In phosphodiester glycans, the methyl group is thought to enter the phosphodiester group, thus neutralizing the negative charge (F).

Esterification [52] and amidation [53,54] are used to derivatize the carboxyl group of sialic acids. These methods improve sialylated glycan detection sensitivity and prevent desialylation during ionization in MS. They neutralize the negative charge of the sialylated glycans, leaving only the negatively charged minor acidic glycans (Fig. 4A∼F). Therefore, when a mixture of sialic acid-derivatized glycans is introduced into the AEC instrument, only minor acidic glycans are retained in the stationary phase. Although several studies have examined sialic acid protection reactions, there are also reports of reactions in which the sialic acid added to the C-3 of galactose is not protected [31,53]. This occurs because the carboxyl group in the sialic acid bound to the C-3 of galactose forms a lactone with the hydroxyl group of galactose. Incomplete blocking of sialic acid thus prevents the selective enrichment of minor acidic glycans. Therefore, care must be taken when selecting the conditions for sialic acid protection.

A method to analyze minor acidic glycans using 3-methyl-1-p-tolyltriazene (MTT)-based sialic acid methylation has been reported [55]. In this method, the glycans released from the proteins are captured in a hydrazine-immobilized resin; the sialic acid on the glycans attached to the resin is then methylated using 3-methyl-1-p-tolyltriazene (MTT). After the methylation of sialic acid, the glycans are released from the resins, and their reducing ends are labeled with benzyloxyamine (BOA) to improve their detection sensitivity under MALDI–TOF MS [56]. These BOA-labeled glycans are examined via AEC, and the minor acidic glycans are enriched. Methylation via MTT causes only one of the two OH groups of the phosphate group to be methylated. Therefore, in contrast to permethylation, MTT-based methylation preserves the negative charge of the phosphate group, enabling the phosphorylated glycans to be enriched via AEC. The introduction of a methyl group into the phosphate group makes it possible to distinguish between the sulfate and phosphate groups using MS. In contrast, this method is unable to detect phosphodiester glycans that have GlcNAc or other substances at the end of the phosphate group. This may be because a methyl group is introduced into the phosphate group of phosphodiester glycans, neutralizing their negative charge [56]. This method has been used to analyze sulfated and phosphorylated glycans in the egg whites of 72 waterfowl species [57] (Table 1); the waterfowl were separated into three groups based on the expression of the sulfated and phosphorylated *N*-glycans. The waterfowl, which are susceptible to avian influenza virus infection, exhibited high expression of phosphorylated hybrid and high-mannose *N*-glycans [57].

A method that uses acetohydrazide to amidate the carboxyl groups of the sialic acid attached to galactose C-3, making it difficult to introduce a protective group, has been developed [54]. The same team has developed a method to selectively enrich sulfated glycopeptides via amidation and AEC [58]. Amidation of glycopeptides neutralizes the negative charges of the carboxyl groups of aspartic acid, glutamic acid, and the C-terminal amino acids, in the same way that those of sialic acid are neutralized. Along with trypsin digestion, this process involves carboxypeptidase digestion to recover the glycopeptide mixture and remove basic amino acids such as lysine from the C-terminus [58]. This process makes it difficult for the carrier peptide of the sulfated glycan to carry a positive charge, thus preventing neutralization of the charges within the molecule. By subjecting this derivatized peptide mixture to AEC, the sulfated glycopeptides can be enriched without being affected by the peptide charge [58]. The enriched sulfated glycopeptides are then measured via MALDI–TOF MS, using sialidase to remove the sialic acid in order to focus on the sulfated glycopeptides; this approach can also be applied to glycopeptides containing sialic acid, and can certainly be applied to native glycans, but not permethylated glycans. This method enables identification of carrier proteins in sulfated glycans [58]. Using this method, *N*-glycans with sulfated LacdiNAc (HSO_3_–4GalNAcGlcNAc-R) were detected on the proteins secreted by human small cell lung cancer (SCLC) cells (Table 1), and their carrier proteins were identified [59].

The sialic acid derivatization method is not considered suitable for the enrichment of glucuronylated glycans. Derivatization reactions of sialic acid occur non-selectively in molecules containing carboxyl groups [58]. Therefore, these reactions are assumed to also occur in the carboxyl group of glucuronic acid, neutralizing its negative charge. In contrast, sulfated glucuronylated glycans, such as the HNK-1 epitope, can be recovered by exploiting the negative charge of the sulfate group.

In the case of phosphate groups, however, the negative charge may or may not be neutralized by sialic acid derivatization, thus preventing the enrichment of phosphorylated glycans. Enrichment of phosphodiester glycans may be similarly difficult. Therefore, when concentrating phosphorylated glycans, it is necessary to consider the conditions of the derivatization reaction.

## 5 Analysis of minor acidic glycans by sialic acid elimination

The easiest way to eliminate the effects of sialic acid is to remove the sialic acid from the glycans. There are two methods of removing sialic acid: neuraminidase digestion [60] and acid hydrolysis [61]. Neuraminidase digestion is used primarily for the analysis of minor acid glycans because it does not degrade other glycans. When a mixture of glycans is digested with neuraminidase from *Arthrobacter ureafaciens*, most of the sialic acids are removed [60], leaving only negatively charged minor acidic glycans. By applying AEC to the resulting mixture of glycans, the sulfated, phosphorylated and glucuronylated minor acidic glycans can be comprehensively enriched. Sialic acid elimination reduces the structural variation owing to sialylation of minor acidic glycans, facilitating their structural analysis and improving their detection sensitivity. Sialic acid elimination is often applied to glycans that are fluorescently labeled via reductive amination. For example, after 2 aminopyridine (PA)-labeled glycans are digested with neuraminidase, they are introduced into diethylaminoethyl (DEAE) cellulose, and the sulfated glycans are enriched [62]. This method was used to analyze sulfated *O*-glycans in human serum from patients with pancreatic and gastric cancer, revealing elevated serum expression of several sulfated *O*-glycans [63]. Sialic acid elimination methods make it possible to analyze the enriched minor acidic glycans using various analytical methods, including fluorescence detection LC and MS. However, the use of DEAE to capture minor acidic glycans requires a large amount of salt for elution, thus necessitating labor-intensive desalting prior to minor acidic glycan analysis.

**Fig. 5:**
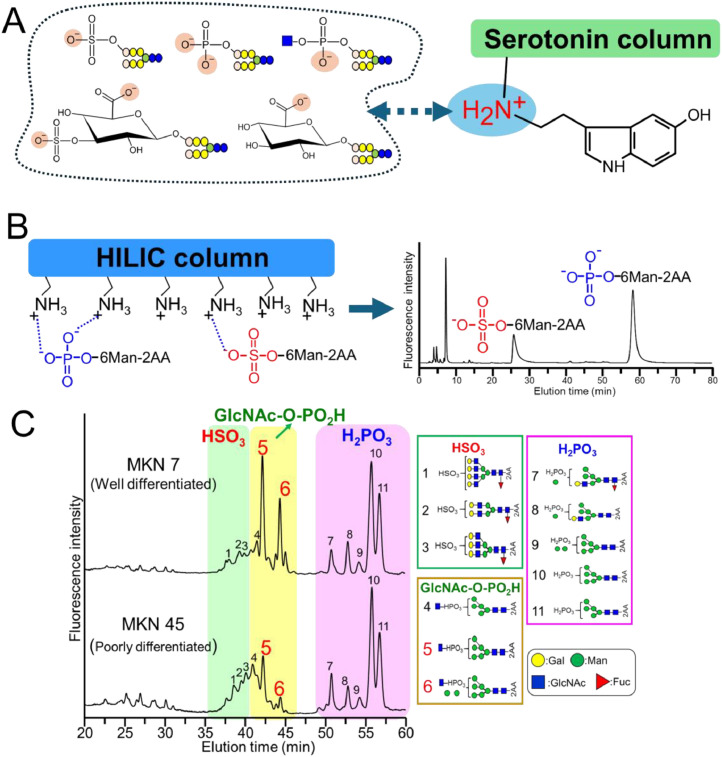
Methods to enrich and separate minor acidic glycans. A) Minor acidic glycan enrichment using a serotonin column. The serotonin's amino group is thought to interact with minor acid glycans. Because this interaction is very weak, the retained minor acidic glycans can be eluted using low-concentration volatile salts. B) Hydrophilic Interaction Liquid Chromatography (HILIC), using an amino column, is used to distinguish between sulfated and phosphorylated glycans. When the mobile phase is weakly acidic, phosphorylated glycans are strongly retained in the stationary phase, because they have one more negative charge than sulfated glycans. When analyzing sulfated and phosphorylated mannose under these conditions, elution times vary substantially. (C) HILIC analysis of minor acidic *N*-glycans from gastric adenocarcinoma cell lines, enriched using a serotonin column and analyzed using an amino column. Under these conditions, sulfated *N*-glycans were observed at 35–41 min, phosphorylated *N*-glycans at 50 min or later, and phosphodiester *N*-glycans at 41–50 min. These figures are based on data published in Ref. [64].

In contrast, we are developing a method to analyze 2-aminobenzoic acid (2AA)-labeled minor acidic glycans [64]. Some studies indicated that 2AA-labeled glycans exhibit stronger fluorescence than PA-labeled or 2-aminobenzamide labeled glycans [36,65], and can also be measured with high sensitivity in negative-ion mode, facilitating MS analysis of acidic glycans. When 2AA-labeled minor acidic glycans are analyzed via negative-ion mode MS/MS, both B and Y ions are detected, making it easy to obtain structural information. To enrich minor acidic glycans, we injected 2AA-labeled glycans digested with neuraminidase into a serotonin column instead of an anion-exchange column (Fig. 5A). Serotonin columns specifically retain sialic acid, while also exhibiting anion-exchange capacity [64,66]. Serotonin contains an amine nitrogen that receives a proton in neutral aqueous solution, becoming positively charged. The amine nitrogen of serotonin is thought to be important for retaining minor acidic glycans.

Serotonin columns exhibit weaker retention than other anion-exchange columns, making it possible to elute the minor acidic glycans trapped in the column using a low concentration of volatile salt. Therefore, when the acidic glycans eluted from the serotonin column evaporate, they can be measured by MS or LC without the need for desalting. However, because serotonin-immobilized columns are difficult to obtain, we have developed a method for the easy enrichment of minor acidic glycans using a spin column and aminopropyl group-immobilized carrier [67]. We have also established a method for group-based separation of minor acidic glycans via Hydrophilic Interaction Liquid Chromatography (HILIC) using an amino column. This HILIC analysis completely separates phosphorylated and sulfated glycans (Fig. 5B). When the sulfate and phosphate groups dissociate in the weakly acidic mobile phase, the phosphate group exhibits a more negative charge than the sulfate group. Therefore, phosphorylated glycans are retained more strongly in the amino column than sulfated glycans and can thus be distinguished from them. We have also detected disulfated glycans and di-phosphodiester glycans in this HILIC condition [64,67]. It is believed that the elution time of glycans slows down as the number of sulfate groups attached to the glycans increases. These methods have been used to analyze minor acidic *N*- and *O*-glycans in various biological samples (Table 1). For example, we have used this method to determine the presence of sulfated *O*-glycans in bovine submaxillary mucin (BSM) [67]. Although the *O*-glycans of bovine submaxillary mucin have been widely analyzed, sulfated *O*-glycans have not previously been detected in this glycoprotein. Our analytical approach is therefore useful for detecting trace amounts of acidic glycans, which have been overlooked in earlier glycomics studies.

**Fig. 6:**
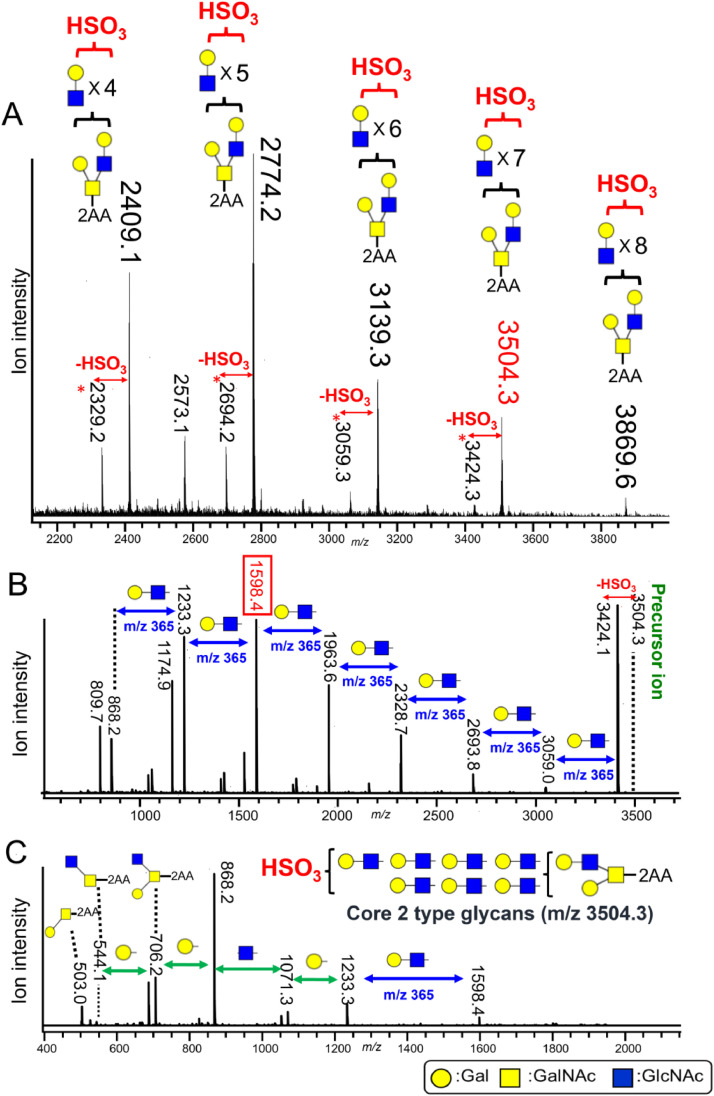
Large sulfated *O*-glycans detected in human gastric adenocarcinoma cells. Minor acidic *O*-glycans prepared from human gastric adenocarcinoma cells, separated using an amino column and analyzed via Matrix-Assisted Laser Desorption/Ionization Quadrupole Ion Trap Time-of-Flight (MALDI–QIT–TOF) Mass Spectrometry (MS) (A). The molecular ion peak at m/z 3504.3, observed in the MS, spectrum was analyzed using MS^2^ (B). The fragment ion peak at m/z 1598.4, observed in the MS/MS spectrum, was analyzed by MS^3^. The figures are taken from Ref. [67].

We have also analyzed the minor acidic *N*-glycans in gastric adenocarcinoma cells at different levels of differentiation (Fig. 5C). Sulfated, phosphorylated, and phosphodiester *N*-glycans were detected when the minor acidic *N*-glycan fractions, prepared from both poorly and well differentiated cells (MKN45 and MKN7, respectively), were separated using an amino column. Interestingly, the phosphodiester *N*-glycan levels (peaks 5 and 6, Fig. 5C) were significantly lower in the poorly differentiated cells than in the well-differentiated cells [64]. When analyzing minor acidic *O*-glycans in the cultured cancer cells, we detected a large polylactosamine-type sulfated *O*-glycan in the MKN45 cells (Fig. 6A). MS/MS analysis of these sulfated glycans revealed the loss of lactosamine residues (Fig. 6B), and the presence of fragment ions of Core 2 *O*-glycans, indicating that they were polylactosamine-type sulfated Core 2 *O*-glycans (Fig. 6C) [67]. Using human colon adenocarcinoma cells (LS174T cells), we also detected 3-deoxy-D-glycero-D-galacto-2-nonulopyranosonic acid (KDN)-containing sulfated *O*-glycans, which have not been previously detected in human cells. Although KDN is a sialic acid, it is resistant to several neuraminidases. We believe that the KDN-containing sulfated *O*-glycans were detected owing to the use of our HILIC-based method utilizing an amino column. These findings reveal that sialic acid elimination is useful for the detection of trace amounts of KDN-containing glycans. Using our proposed method, we also identified phosphorylated ribose, which is thought to bind to serine/threonine residues, in all of the human cancer cells that we examined [67].

**Fig. 7:**
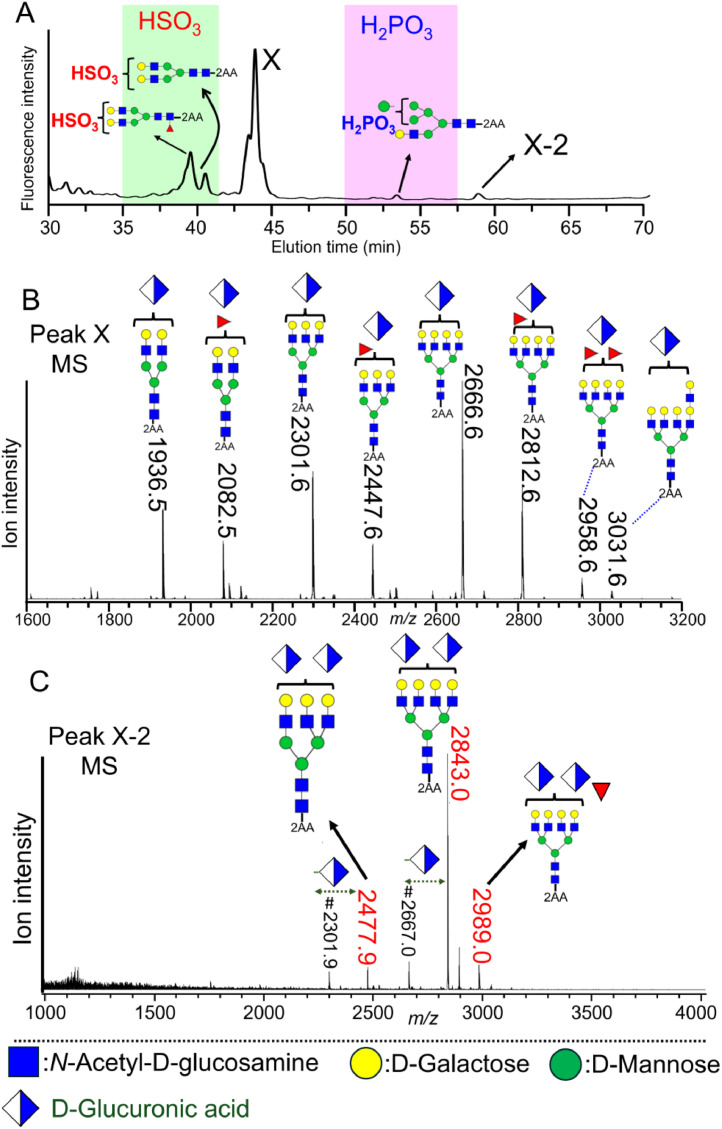
Minor acidic N-glycans detected in human serum. The minor acidic *N*-glycan fraction was analyzed using an amino column (A). Peak X in the chromatogram was analyzed using Matrix-Assisted Laser Desorption/Ionization Quadrupole Ion Trap Time-of-Flight (MALDI–QIT–TOF) mass spectrometry (MS) (B). Peak X represents various monoglucuronylated *N*-glycans. Peak X-2 in the chromatogram was analyzed by MALDI–QIT–TOF MS (C). These figures are modified versions of the data published in [68].

Using our proposed method, we analyzed minor acidic *N*-glycans in human serum [68]. Analysis of the minor acidic *N*-glycan fraction in human serum using an amino column identified sulfated complex and phosphorylated hybrid glycans (Fig. 7A). MS analysis of peak X in the chromatogram revealed that it represents multiple non-sulfated glucuronylated *N*-glycans (Fig. 7B); Peak X2, in contrast, represents diglucuronylated *N*-glycans (Fig. 7C). Our comparison of the expression of these minor acidic glycans in healthy individuals and in patients with pancreatic cancer revealed reduced levels of glucuronylated *N*-glycans in the serum of the patients with pancreatic cancer, potentially reflecting an association between pancreatic cancer and reduced serum glucuronylated *N*-glycan levels. Although *N*-glycans in human serum have been widely analyzed, no prior reports have identified glucuronylated *N*-glycans in human serum. Our analysis therefore provides the first evidence of glucuronylated *N*-glycans in human serum.

Although sialic acid elimination does not provide information on sialylation of glycans, it does facilitate comprehensive analysis of minor acidic glycans. However, when using neuraminidase to remove sialic acid, it is necessary to be aware that some forms of sialic acid are resistant to neuraminidase. Although this resistance may interfere with minor acidic glycans enrichment, sialic acid elimination is useful for detecting rare sialic acids such as KDN. If the intention is to completely eliminate the effects of sialic acid, acid hydrolysis will be more effective than neuraminidase digestion [61]. However, as acid hydrolysis can also degrade minor acidic glycans, it is necessary to adjust the conditions of the hydrolysis reaction according to the measurement target.

## 6 Isolation of target proteins

Minor acidic glycans have been detected via glycan analysis of specific proteins. Supported Molecular Matrix Electrophoresis (SMME), developed to isolate and separate mucins in biological samples, has been used to isolate mucin-rich acidic glycans from porcine stomach mucin, identifying sulfated *O*-glycans on the mucin [69]. In addition, minor acidic glycans are detected in the acidic glycoproteins fractionated using ACE. For example, sulfated O-glycans were detected in acidic glycoproteins purified using ACE from ovarian tumor cyst fluid [70]. DEAE-Sepharose has been also used to purify acidic glycoproteins from mouse brains, and *O*-mannose glycans with the HNK-1 epitope have been identified in these acidic glycoproteins [71]. Therefore, focusing on acidic glycoproteins may be an effective method for analyzing minor acidic glycans.

Sulfated *N*-glycans have been identified in human serum IgG [72]. Following the enrichment of human serum IgG using Protein A-immobilized Sepharose, the *N*-glycans were released from the IgG; the acidic glycans were then enriched on a TiO_2_-PGC chip and were detected via LC-MS. The TiO_2_-PGC chip, a chip-based column for the nano LC system, comprises a TiO_2_ column and a graphite carbon column; the acidic glycans are enriched in the TiO_2_ column and separated in the graphite carbon column. TiO_2_ acts as a specific enrichment carrier for phosphorylated peptides, although its anion-exchange capacity facilitates the enrichment of sialylated and sulfated glycans. It may be possible to enrich minor acidic glycans using the TiO_2_-PGC chip, although it is difficult to separate minor acidic glycans from sialylated glycans [73]. Therefore, if this method is applied to samples containing significantly lower levels of minor acid glycan than sialylated glycans, it may be necessary to perform pretreatment, such as via purification of specific glycoproteins.

Analysis of the *O*-glycans in α-dystroglycans, an important area of research, has identified phosphorylated *O*-mannose and phosphodiester *O*-mannose glycans, with ribitol or glycerol attached to the phosphate groups [18]. Moreover, new minor acidic glycans have been discovered via targeting of specific proteins followed by the analysis of minor acidic glycans.

## 7 Conclusions

The novel methods summarized herein can be used to identify glycans that are difficult to detect using the existing comprehensive glycomics and glycoproteomics methods. These methods make it possible to identify and elucidate previously overlooked relationships between glycans and biological phenomena. Such methods are expected to lead to the discovery of novel glycan markers.

The various methods for minor acid glycan analysis each have various advantages and disadvantages, and a comprehensive method is yet to be established. Therefore, it is necessary to select the optimal method according to the available laboratory equipment and the objectives of the analysis. Efforts should be made to develop a more complete method to analyze minor acidic glycans. To achieve this, more research on minor acidic glycans in biological samples, using previously established methods, is required. Clarifying the distribution of minor acidic glycans in biological samples will help in optimizing the analytical methods and in developing more efficient methods. Determining the location of the sulfate, phosphate, and glucuronic acid groups in the glycan remains challenging. In some cases, the binding-position can be identified through MS/MS analysis, although it is difficult to characterize the structures of trace components using this method. In addition, MS analysis of minor acidic glycans becomes challenging following the loss of acidic groups due to post-source or in-source decay. Future work is expected to yield new methods for determining the detailed structure of minor acidic glycans.

## Funding

This study was supported by JSPS KAKENHI [grant numbers 23K06075 and 20K07004] and the Assisted Joint Research Program (Exploration Type) of the J-GlycoNet cooperative network, accredited by the Minister of Education, Culture, Sports, Science, and Technology, MEXT, Japan, as a Joint Usage/Research Center [JSPS Core-to-Core Program No. JPJSCCA202000.

## CRediT authorship contribution statement

**Keita Yamada:** Writing – review & editing, Writing – original draft, Conceptualization.

## Declaration of competing interest

The authors declare that they have no known competing financial interests or personal relationships that could have appeared to influence the work reported in this paper.

## Data Availability

Data will be made available on request.

## References

[1] Marciel M.P., Haldar B., Hwang J., Bhalerao N., Bellis S.L. (2023). Role of tumor cell sialylation in pancreatic cancer progression. Adv. Cancer Res..

[2] Xu C., Wang S., Wu Y., Sun X., Yang D., Wang S. (2021). Recent advances in understanding the roles of sialyltransferases in tumor angiogenesis and metastasis. Glycoconj. J..

[3] Furukawa J.I., Hanamatsu H., Nishikaze T., Manya H., Miura N., Yagi H., Yokota I., Akasaka-Manya K., Endo T., Kanagawa M., Iwasaki N., Tanaka K. (2020). Lactone-driven ester-to-amide derivatization for sialic acid linkage-specific alkylamidation. Anal. Chem..

[4] Kobayashi M., Fukuda M., Nakayama J. (2009). Role of sulfated O-glycans expressed by high endothelial venule-like vessels in pathogenesis of chronic inflammatory gastrointestinal diseases. Biol. Pharm. Bull..

[5] Patnode M.L., Yu S.Y., Cheng C.W., Ho M.Y., Tegesjö L., Sakuma K., Uchimura K., Khoo K.H., Kannagi R., Rosen S.D. (2013). KSGal6ST generates galactose-6-O-sulfate in high endothelial venules but does not contribute to L-selectin-dependent lymphocyte homing. Glycobiology.

[6] Lo-Guidice J.M., Périni J.M., Lafitte J.J., Ducourouble M.P., Roussel P., Lamblin G. (1995). Characterization of a sulfotransferase from human airways responsible for the 3-O-sulfation of terminal galactose in N-acetyllactosamine-containing mucin carbohydrate chains. J. Biol. Chem..

[7] Wang R., Wu X.Z. (2014). Roles of galactose 3′-O- sulfation in signaling. Glycoconj. J..

[8] Hirano K., Furukawa K. (2022). Biosynthesis and biological significances of LacdiNAc group on N- and O-glycans in human cancer cells. Biomolecules.

[9] Yamashita K., Ueda I., Kobata A. (1983). Sulfated asparagine-linked sugar chains of hen egg albumin. J. Biol. Chem..

[10] Ertunc N., Phitak T., Wu D., Fujita H., Hane M., Sato C., Kitajima K. (2022). Sulfation of sialic acid is ubiquitous and essential for vertebrate development. Sci. Rep..

[11] Kobayashi M., Mitoma J., Nakamura N., Katsuyama T., Nakayama J., Fukuda M. (2004). Induction of peripheral lymph node addressin in human gastric mucosa infected by Helicobacter pylori. Proc. Natl Acad. Sci. U. S. A..

[12] Suzawa K., Kobayashi M., Sakai Y., Hoshino H., Watanabe M., Harada O., Ohtani H., Fukuda M., Nakayama J. (2007). Preferential induction of peripheral lymph node addressin on high endothelial venule-like vessels in the active phase of ulcerative colitis. Am. J. Gastroenterol..

[13] Coutinho M.F., Prata M.J., Alves S. (2012). Mannose-6-phosphate pathway: a review on its role in lysosomal function and dysfunction. Mol. Genet. Metab..

[14] Kornfeld R., Bao M., Brewer K., Noll C., Canfield W. (1999). Molecular cloning and functional expression of two splice forms of human N-acetylglucosamine-1-phosphodiester alpha-N-acetylglucosaminidase. J. Biol. Chem..

[15] Braulke T., Carette J.E., Palm W. (2024). Lysosomal enzyme trafficking: from molecular mechanisms to human diseases. Trends Cell Biol.

[16] Yoshida-Moriguchi T., Yu L., Stalnaker S.H., Davis S., Kunz S., Madson M., Oldstone M.B., Schachter H., Wells L., Campbell K.P. (2010). O-mannosyl phosphorylation of alpha-dystroglycan is required for laminin binding. Science.

[17] Endo T. (2015). Glycobiology of α-dystroglycan and muscular dystrophy. J. Biochem..

[18] Yagi H., Kuo C.W., Obayashi T., Ninagawa S., Khoo K.H., Kato K. (2016). Direct mapping of additional modifications on phosphorylated O-glycans of α-dystroglycan by mass spectrometry analysis in conjunction with knocking out of causative genes for dystroglycanopathy. Mol. Cell. Proteomics..

[19] Chittum J.E., Thompson A., Desai U.R. (2024). Glycosaminoglycan microarrays for studying glycosaminoglycan–protein systems. Carbohydr. Polym..

[20] Abo T., Balch C.M. (1981). A differentiation antigen of human NK and K cells identified by a monoclonal antibody (HNK-1). J. Immunol (Baltimore, Md.: 1950).

[21] Kleene R., Schachner M. (2004). Glycans and neural cell interactions. Nat. Rev. Neurosci..

[22] Morise J., Takematsu H., Oka S. (2017). The role of human natural killer-1 (HNK-1) carbohydrate in neuronal plasticity and disease. Biochim. Biophys. Acta Gen. Subj..

[23] García-Ayllón M.S., Botella-López A., Cuchillo-Ibañez I., Rábano A., Andreasen N., Blennow K., Ávila J., Sáez-Valero J. (2017). HNK-1 carrier glycoproteins are decreased in the Alzheimer's disease brain. Mol. Neurobiol..

[24] Tagawa H., Kizuka Y., Ikeda T., Itoh S., Kawasaki N., Kurihara H., Onozato M.L., Tojo A., Sakai T., Kawasaki T., Oka S. (2005). A non-sulfated form of the HNK-1 carbohydrate is expressed in mouse kidney. J. Biol. Chem..

[25] Suttapitugsakul S., Stavenhagen K., Donskaya S., Bennett D.A., Mealer R.G., Seyfried N.T., Cummings R.D. (2022). Glycoproteomics landscape of asymptomatic and symptomatic human Alzheimer's disease brain. Mol. Cell. Proteomics..

[26] Narimatsu H., Sawaki H., Kuno A., Kaji H., Ito H., Ikehara Y. (2010). A strategy for discovery of cancer glyco-biomarkers in serum using newly developed technologies for glycoproteomics. FEBS J.

[27] Markandran K., Xuan J.V.L.E., Yu H., Shun L.M., Ferenczi M.A. (2021). Mn^2+^ -Phos-tag polyacrylamide for the quantification of protein phosphorylation levels. Curr. Protoc..

[28] Plummer T.H., Tarentino A.L. (1991). Purification of the oligosaccharide-cleaving enzymes of Flavobacterium meningosepticum. Glycobiology.

[29] Nakakita S., Sumiyoshi W., Miyanishi N., Hirabayashi J. (2007). A practical approach to N-glycan production by hydrazinolysis using hydrazine monohydrate. Biochem. Biophys. Res. Commun..

[30] Ishibashi Y., Kobayashi U., Hijikata A., Sakaguchi K., Goda H.M., Tamura T., Okino N., Ito M. (2012). Preparation and characterization of EGCase I, applicable to the comprehensive analysis of GSLs, using a rhodococcal expression system. J. Lipid Res..

[31] Pintado-Sierra M., García-Álvarez I., Bribián A., Medina-Rodríguez E.M., Lebrón-Aguilar R., Garrido L., de Castro F., Fernández-Mayoralas A., Quintanilla-López J.E. (2017). A comprehensive profiling of sulfatides in myelin from mouse brain using liquid chromatography coupled to high-resolution accurate tandem mass spectrometry. Anal. Chim. Acta..

[32] Kameyama A., Thet Tin W.W., Toyoda M., Sakaguchi M. (2019). A practical method of liberating O-linked glycans from glycoproteins using hydroxylamine and an organic superbase. Biochem. Biophys. Res. Commun..

[33] Yamada K., Hirabayashi J., Kakehi K. (2013). Analysis of O-glycans as 9-fluorenylmethyl derivatives and its application to the studies on glycan array. Anal. Chem..

[34] Schulz B.L., Packer N.H., Karlsson N.G. (2002). Small-scale analysis of O-linked oligosaccharides from glycoproteins and mucins separated by gel electrophoresis. Anal. Chem..

[35] Kozak R.P., Royle L., Gardner R.A., Bondt A., Fernandes D.L., Wuhrer M. (2014). Improved nonreductive O-glycan release by hydrazinolysis with ethylenediaminetetraacetic acid addition. Anal. Biochem..

[36] Kinoshita M., Yamada K. (2022). Recent advances and trends in sample preparation and chemical modification for glycan analysis. J. Pharm. Biomed. Anal..

[37] Hu Z., Liu R., Gao W., Li J., Wang H., Tang K. (2024). A fully automated online enrichment and separation system for highly reproducible and in-depth analysis of intact glycopeptide. Anal. Chem..

[38] Rafique S., Yang S., Sajid M.S., Faheem M. (2024). A review of intact glycopeptide enrichment and glycan separation through hydrophilic interaction liquid chromatography stationary phase materials. J. Chromatogr. A..

[39] Chen J.-Y., Huang H.-H., Yu S.-Y., Wu S.-J., Kannagi R., Khoo K.-H. (2018). Concerted mass spectrometry-based glycomic approach for precision mapping of sulfo sialylated N-glycans on human peripheral blood mononuclear cells and lymphocytes. Glycobiology.

[40] Mechref Y., Kang P., Novotny M.V. (2009). Solid-phase permethylation for glycomic analysis. Methods Mol. Biol..

[41] Lei M., Mechref Y., Novotny M.V. (2009). Structural analysis of sulfated glycans by sequential double-permethylation using methyl iodide and deuteromethyl iodide. J. Am. Soc. Mass Spectrom..

[42] Lei M., Novotny M.V., Mechref Y. (2010). Sequential enrichment of sulfated glycans by strong anion-exchange chromatography prior to mass spectrometric measurements. J. Am. Soc. Mass Spectrom..

[43] Cheng C.-W., Chou C.-C., Hsieh H.-W., Tu Z., Lin C.-H., Nycholat C., Fukuda M., Khoo K.-H. (2015). Efficient mapping of sulfated glycotopes by negative ion mode nanoLC–MS/MS-based Sulfoglycomic analysis of permethylated glycans. Anal. Chem..

[44] Cheng P.-F., Snovida S., Ho M.-Y., Cheng C.-W., Wu A.M., Khoo K.-H. (2013). Increasing the depth of mass spectrometry-based glycomic coverage by additional dimensions of sulfoglycomics and target analysis of permethylated glycans. Anal. Bioanal. Chem..

[45] Hsiao C.-T., Wang P.-W., Chang H.-C., Chen Y.-Y., Wang S.-H., Chern Y., Khoo K.-H. (2017). Advancing a high throughput glycotope-centric glycomics workflow based on nanoLC-MS2-product dependent-MS3 ANAlysis of permethylated glycans. Mol. Cell. Proteomics..

[46] Hoshino H., Chen Y.-Y., Inoue D., Yoshida Y., Khoo K.-H., Akama T.O., Kobayashi M. (2024). Expression of low-sulfated keratan sulfate in non-mucinous ovarian carcinoma. Glycobiology.

[47] Inoue D., Hoshino H., Chen Y.-Y., Yamamoto M., Kogami A., Fukushima M., Khoo K.-H., Akama T.O., Yoshida Y., Kobayashi M. (2024). Structural elucidation and prognostic relevance of 297–11A-sulfated glycans in ovarian carcinoma. Lab. Invest..

[48] Tseng H.C., Hsiao C.T., Yamakawa N., Guérardel Y., Khoo K.H. (2021). Discovery sulfoglycomics and identification of the characteristic fragment ions for high-sensitivity precise mapping of adult zebrafish brain-specific glycotopes. Front. Mol. Biosci..

[49] Kumagai T., Katoh T., Nix D.B., Tiemeyer M., Aoki K. (2013). In-gel β-elimination and aqueous–organic partition for improved O- and sulfoglycomics. Anal. Chem..

[50] Kurz S., Aoki K., Jin C., Karlsson N.G., Tiemeyer M., Wilson I.B., Paschinger K. (2015). Targeted release and fractionation reveal glucuronylated and sulphated N- and O-glycans in larvae of dipteran insects. J. Proteomics..

[51] Dutta S., Aoki K., Doungkamchan K., Tiemeyer M., Bovin N., Miller D.J. (2019). Sulfated Lewis A trisaccharide on oviduct membrane glycoproteins binds bovine sperm and lengthens sperm lifespan. J. Biol. Chem..

[52] Miura Y., Shinohara Y., Furukawa J., Nagahori N., Nishimura S.I. (2007). Rapid and simple solid-phase esterification of sialic acid residues for quantitative glycomics by mass spectrometry. Chemistry.

[53] Sekiya S., Wada Y., Tanaka K. (2005). Derivatization for stabilizing sialic acids in MALDI-MS. Anal. Chem..

[54] Toyoda M., Ito H., Matsuno Y.K., Narimatsu H., Kameyama A. (2008). Quantitative derivatization of sialic acids for the detection of sialoglycans by MALDI MS. Anal. Chem..

[55] Montalban B.M., Hinou H. (2023). Glycoblotting enables seamless and straightforward workflow for MALDI-TOF/MS-based sulphoglycomics of N- and O-glycans. Proteomics.

[56] Gebrehiwot A.G., Melka D.S., Kassaye Y.M., Rehan I.F., Rangappa S., Hinou H., Kamiyama T., Nishimura S.I. (2018). Healthy human serum N-glycan profiling reveals the influence of ethnic variation on the identified cancer-relevant glycan biomarkers. PLOS ONE.

[57] Montalban B.M., Hinou H. (2024). Glycoblotting-based ovo-sulphoglycomics reveals phosphorylated N-glycans as a possible Host Factor of AIV Prevalence in Waterfowls. ACS Infect. Dis..

[58] Toyoda M., Narimatsu H., Kameyama A. (2009). Enrichment method of sulfated glycopeptides by a sulfate emerging and ion exchange chromatography. Anal. Chem..

[59] Toyoda M., Kaji H., Sawaki H., Togayachi A., Angata T., Narimatsu H., Kameyama A. (2016). Identification and characterization of sulfated glycoproteins from small cell lung carcinoma cells assisted by management of molecular charges. Glycoconj. J..

[60] Prime S., Dearnley J., Ventom A.M., Parekh R.B., Edge C.J. (1996). Oligosaccharide sequencing based on exo- and endoglycosidase digestion and liquid chromatographic analysis of the products. J. Chromatogr. A..

[61] Iwatsuka K., Yasueda S., Bando E., Fujii H., Terada T., Okubo H., Iwamoto H., Kinoshita M., Kakehi K. (2011). Comparative studies of HPLC-fluorometry and LC/MS method for the determination of N-acetylneuraminic acid as a marker of deteriorated ophthalmic solutions. J. Chromatogr. B Analyt. Technol. Biomed. Life Sci..

[62] Yagi H., Takahashi N., Yamaguchi Y., Kimura N., Uchimura K., Kannagi R., Kato K. (2005). Development of structural analysis of sulfated N-glycans by multidimensional high performance liquid chromatography mapping methods. Glycobiology.

[63] Tanaka-Okamoto M., Mukai M., Takahashi H., Fujiwara Y., Ohue M., Miyamoto Y. (2017). Various sulfated carbohydrate tumor marker candidates identified by focused glycomic analyses. Glycobiology.

[64] Yamada K., Kayahara H., Kinoshita M., Suzuki S. (2018). Simultaneous analysis of sulfated and phosphorylated glycans by serotonin-immobilized column enrichment and hydrophilic interaction chromatography. Anal. Chem..

[65] Kim W., Kim J., You S., Do J., Jang Y., Kim D., Lee J., Ha J., Kim H.H. (2019). Qualitative and quantitative characterization of sialylated N-glycans using three fluorophores, two columns, and two instrumentations. Anal. Biochem..

[66] Yamada K., Mitsui Y., Kakoi N., Kinoshita M., Hayakawa T., Kakehi K. (2012). One-pot characterization of cancer cells by the analysis of mucin-type glycans and glycosaminoglycans. Anal. Biochem..

[67] Yamada K., Asada K., Hanzawa K., Aoki Y., Nakajima K., Kinoshita M. (2024). Developing method for minor acidic O-glycan analysis in mucin and cancer cell samples. J. Proteome Res..

[68] Yamada K., Suzuki K., Hirohata Y., Kinoshita M. (2020). Analysis of minor acidic N-glycans in human serum. J. Proteome Res..

[69] Matsuno Y.K., Saito T., Gotoh M., Narimatsu H., Kameyama A. (2009). Supported molecular matrix electrophoresis: a new tool for characterization of glycoproteins. Anal. Chem..

[70] Thomsson K.A., Vitiazeva V., Mateoiu C., Jin C., Liu J., Holgersson J., Weijdegård B., Sundfeldt K., Karlsson N.G. (2021). Sulfation of O-glycans on mucin-type proteins from serous ovarian epithelial tumors. Mol. Cell. Proteomics..

[71] Morise J., Kizuka Y., Yabuno K., Tonoyama Y., Hashii N., Kawasaki N., Manya H., Miyagoe-Suzuki Y., Takeda S., Endo T., Maeda N., Takematsu H., Oka S. (2014). Structural and biochemical characterization of O-mannose-linked human natural killer-1 glycan expressed on phosphacan in developing mouse brains. Glycobiology.

[72] Wang J.R., Gao W.N., Grimm R., Jiang S., Liang Y., Ye H., Li Z.G., Yau L.F., Huang H., Liu J., Jiang M., Meng Q., Tong T.T., Huang H.H., Lee S., Zeng X., Liu L., Jiang Z.H. (2017). A method to identify trace sulfated IgG N-glycans as biomarkers for rheumatoid arthritis. Nat. Commun.

[73] Yau L.-F., Chan K.-M., Yang C.-G., Ip S.-W., Kang Y., Mai Z.-T., Tong T.-T., Jiang Z.-H., Yang Z.-F., Wang J.-R. (2020). Comprehensive glycomic profiling of respiratory tract tissues of tree shrews by TiO_2_-PGC chip mass spectrometry. J. Proteome Res..

